# Salivary Alpha-Amylase in Experimentally-Induced Muscle Pain

**DOI:** 10.3390/diagnostics10090722

**Published:** 2020-09-20

**Authors:** Nikolaos Christidis, Pegah Baghernejad, Aylin Deyhim, Hajer Jasim

**Affiliations:** Division of Oral Diagnostics and Rehabilitation, Department of Dental Medicine Karolinska Institutet and Scandinavian Center for Orofacial Neurosciences (SCON), SE 14104 Huddinge, Sweden; nikolaos.christidis@ki.se (N.C.); pegah.ba@hotmail.com (P.B.); aylindeyhim@hotmail.se (A.D.)

**Keywords:** salivary alpha-amylase, craniofacial pain, saliva, stress

## Abstract

Salivary alpha-amylase (sAA) is a marker of psychological stress and might also be a potential marker for pain-associated stress due its non-invasive, cost-effective, and stress-free collection. The current study aimed to investigate whether the levels of sAA are influenced by experimentally induced muscle pain. In this study, 26 healthy, pain-free and age-matched participants (23.8 ± 2.6 years) were included, 13 women and 13 men. Prior to the experiment, questionnaires assessing health and anxiety were completed. Muscle pain was then induced through intramuscular injection of 0.4 mL hypertonic saline (56.5 mg/mL) into the masseter muscle and unstimulated whole saliva samples were collected at baseline before injection, 2 min, and 15 min after injection. A commercially available colorimetric assay was used to analyze the sAA. Perceived pain and stress were assessed using a 0–100 Numeric Rating Scale for each sample. There were no significant differences in sAA levels prior and after injection of hypertonic saline (*p* > 0.05) although sAA levels showed a slight decrease during experimentally-induced muscle pain. However, a strong correlation was observed between self-reported pain and perceived level of stress during experimentally-induced muscle pain (r_2_ = 0.744; *p* < 0.0001). Furthermore, there was a moderate correlation between the levels of sAA at baseline and during experimental pain (r_2_ = 0.687; *p* < 0.0001). In conclusion, this study could not show any association between the levels of sAA and perceived pain and or/stress. However, since a significant strong correlation could be observed between perceived stress and pain intensity, this study indicates that experimentally-induced muscle pain could be used as a stress model.

## 1. Introduction

Saliva is produced by three major glands and several minor accessory glands. Human saliva has several important functions, such as moistening and lubricating the oral tissues and functioning as a taste solvent and a swallowing agent. Salivary alpha amylase (sAA) is one of the most essential enzymes in saliva and is mostly synthesized by the parotid gland [1,2,3,4].

Secretion of alpha amylase from the salivary glands is controlled by autonomic nervous system (ANS) and substantial literature reveals that secretion of sAA seems to correlate to sympathetic activity under conditions of stress [3,5,6,7,8,9]. The first study examining sAA levels in subjects being exposed to hyperbaric pressure during several days, by Gilman et al., showed a higher concentration of sAA in saliva [10]. This and numerous other studies since then have suggested that these increases are due to the hyperbaric pressure and the effect it has on ANS, but also due to psychological stress that the subjects are exposed to, indicating that sAA could be a non-invasive marker for psychological stress due to the activation of the sympathetic-adreno-medullar (SAM) system [3,5,6,7,8,11,12,13,14,15,16]. Based on this, one can assume that sAA is a valuable marker for assessing sympathetic activation. However, the validity still remains debatable since sAA activity does not always strongly correlate with sympathetic activation [17]. Since painful stimuli activate two major stress response systems, one of them being the SAM system, sAA might also reflect on pain associated stress levels [6,7,8,9,11,18,19,20,21,22,23]. Previous studies have shown that sAA has a direct response to a stressful stimulus [24], shorter latency time to peak level, and no carry-over effect compared to cortisol [21,22,23]. Based on this, and since some studies have also shown significant correlations between self-reported pain and levels of sAA, it is likely that sAA could serve as a potential objective non-invasive tool for pain assessments [21,22,23].

Self-reported pain is important for adequate therapy, but in some cases, where patients are unable to subjectively assess pain, an objective method would be preferable. Saliva sampling has a lot of advantages: it is easy to collect, non-invasive, cost-effective, and stress-free [25]. Salivary samples can, therefore, be collected without being contingent on any medical staff, which makes it uncomplicated and accessible. In addition to the easy procedure of sampling, it is also easily stored. Saliva samples offer a non-invasive method in contrast to other sampling methods, e.g., blood or cerebrospinal fluid. These procedures might also be accompanied by anxiety or pain, which are stressors themselves [25,26,27]. Despite the advantages, there are a few drawbacks that need to be considered. sAA levels are not specifically only triggered by pain; other stressors also have an impact on sAA activation, such as exercise and physical and psychosocial stress [17]. A study by Takai et al. showed that sAA levels correlate to perceived anxiety [16]. sAA levels also vary among humans due to the human salivary amylase gene and are positively correlated to sAA protein levels [28]. Activation of a stress marker such as sAA offers the opportunity to collect data from nonverbal or preverbal toddlers, geriatric patients, infants, and patients with cognitive restrictions or even anxious or sensitive patients in a context that would indicate pain. This would be very valuable and could lead to less suffering, as well as more accurate and/or individualized pain therapy.

The aim of this study was to investigate if the experimentally-induced muscle pain influences the level of sAA, and if the levels of sAA correlates with the pain intensity. The hypothesis was that experimentally-induced muscle pain will affect the ANS and consequently result in increased secretion of sAA.

## 2. Methods and Materials

### 2.1. Participants

Twenty-six healthy participants, 13 men and 13 age-matched women, were included in the study, with a mean ± SD age of 23.8 ± 2.6 years. The participants were recruited through an advertisement on social media and among undergraduate dental students at Karolinska Institutet, Huddinge, Sweden.

Inclusion criteria were: (a) good general health; (b) age ≥ 18 years; and (c) body mass index <30 kg/m^2^. Participants also had to maintain exceptional oral hygiene on the day of collection.

Exclusion criteria were: (1) any current pain; (2) a pain-related diagnosis according to the diagnostic criteria for temporomandibular disorders (DC/TMD) [14]; (3) a diagnosed systemic muscular or joint disease; (4) whiplash-associated disorders; (5) neurological or neuropsychiatric disorders; (6) pregnancy or lactation; (7) high blood pressure; (8) use of any medication; (9) oral complaints; and (10) tobacco usage.

All participants received information regarding the objectives and procedures of the study and gave their informed written consent before the start of the experiment. The study protocol was approved by the Swedish Ethical Review Authority, Sweden (2019–00494, 18 February 2019) and followed Good Clinical Practice and the guidelines according to the Helsinki declaration.

### 2.2. Study Design

All participants who fulfilled the inclusion criteria underwent a clinical examination according to the Swedish version of the DC/TMD axis I [29]. The evidence-based protocol was used as a screening instrument for identification of participants with TMD signs. Participants showing clinical signs of TMD (except for disc displacement without reduction, which was considered as normal fluctuation) were excluded from further involvement in the experiment.

Prior to saliva collection, participants were instructed to fill in validated questionnaires to assess the degree of depression, somatic symptoms, and current and general anxiety. The perceived health questionnaires (PHQ) were used to assess anxiety (PHQ-9) and somatization (PHQ-15). PHQ-9 consisting of nine questions regarding depression, asking how often over the past two weeks the participants have been bothered by various feelings related to depressive symptoms, with four possible answers 0 (not bothered), 1 (several days), 2 (more than half of the days), and 3 (almost every day). Scores ranged between 0 and 27, and scores of 5, 10, 15, and 20 were considered cut-off values for mild, moderate, moderately severe, and severe depression, respectively. Studies of the reliability and validity in adults shows that the PHQ-9 has a 61% sensitivity and 94% specificity [30,31]. The PHQ-15 is a somatic instrument including 15 somatic symptoms or symptom clusters that account for more than 90% of the physical complaints. In determining the PHQ-15 score, each symptom is coded as 0, 1, or 2, and the total score ranges from 0 to 30. Scores of 5, 10, and 15 are considered cut-off values for mild, moderate, and severe somatic symptoms, respectively [32,33]. The State-Trait and STAI-Strait Anxiety Inventory (STAI) is a validated useful instrument to assess mental health. The STAI-Strait consists of questions regarding how the participants feel at the moment, and the STAI-Trait describes how the patients feel in general. It consists of 20 questions with a range of four possible answers representing each types of anxiety, 1 (no), 2 (very little), 3 (pretty high), and 4 (very high). Total scores range from 20 to 80, with the higher scores representing a correlation with greater anxiety [16].

The experiment began by randomizing the side for intramuscular injection of hypertonic saline. During the study, unstimulated whole saliva was collected at three time points: at baseline before induced pain, during experimentally-induced muscle pain, and 15 min after the induced pain for measurement of the sAA activity. Before each saliva sampling, the perceived pain and stress were assessed using a Numeric Rating Scale (NRS). The scale range was from 0–100, where 0 was no stress or pain experienced and 100 was maximal stress or worst pain experienced.

After the first saliva sample was collected, muscle pain was induced by an intramuscular injection of hypertonic saline 0.4 mL (56.5 mg/mL) into the prominent part of the masseter muscle during approximately 30 s. One minute after the injection the participant assessed perceived pain and stress on a 0–100 NRS. A second saliva sample was collected for another 3 min and the participants were asked again to assess the perceived pain and stress on the NRS. The third and last saliva sample was collected 15 min after the injection and the participants were asked to once more assess the perceived pain and stress on the NRS. They were also asked to assess how stressed they felt during the whole experiment using the Rated Perceived Exertion (RPE) graded from 0 (no perceived stress) to 10 (maximum perceived stress). The Rated Perceived Exertion scale was used to measure the experienced exertion [34].

### 2.3. Saliva Collection

The participants were instructed not to eat, drink, or brush their teeth a minimum of one hour prior to sample collection. To avoid variation of sAA secretion due to the diurnal changes during the day, most of the samples were collected in early afternoon.

Unstimulated whole saliva was collected while the participants were seated comfortably with eyes open and head slightly tilted forward, as previously illustrated by Jasim et al. [25]. Participants were instructed to allow saliva to accumulate on the floor of the mouth without stimulation and passively drool into a polypropylene tube. Saliva was collected for a total time of 3 min, at three time points—prior, 1 min after injection, and finally 15 min after injection. The samples were directly stored at −20 °C until analysis.

### 2.4. Salivary Alpha-Amylase

Saliva samples were thawed, vortexed, and centrifuged at 1500× *g* for 15 min in order to remove mucins and debris. All samples were diluted and assayed in duplicate in order to reduce the margin of error. sAA activity were measured using a commercially available enzymatic assay kit according the instructions from the manufacturer (Salimetrics, State College, PA, USA).

### 2.5. Statistics

The sample size was based on a power calculation showing that 25 participants were needed to be able to reject the null hypothesis, given a significance level of *p* < 0.05, a power of 80%, an estimated mean difference of 35%, and a standard deviation of 60% (based on a previous study not yet published).

The Shapiro-Wilks test was used to study the distribution of the data. Differences in descriptive variables between males and females in the study were tested with the Mann-Whitney *U*-test since most variables did not show normal distribution. Analysis of variance (Friedman’s ANOVA) was used for repeated measurements to analyze the variation of sAA, perceived stress, and pain over time. When significant, post-hoc analysis with Wilcoxon matched pair-test was applied and adjusted for multiple comparisons according to Bonferroni.

The Spearman correlation test was used to test for significant correlations between variables with additional Bonferroni adjustments. Descriptive data are presented as mean ± SD or median and interquartile range (IQR). For all analyses, the significance level was set at *p* < 0.05. The statistical analyses were performed using Statistica version 13 (StatSoft, Tulsa, OK, USA).

## 3. Results

### 3.1. Data Overview

Descriptive data of the participants (*n* = 26) included in the experiment are presented in Table 1. Males and females were similar in background factors and reported similar levels of psychological factors (*p* > 0.05), which is why data further on are presented on a group level.

sAA levels showed no significant differences between sexes at baseline or after injection. However, the sAA level 15 min after injection was significantly higher in males compared to females (Z = −2.09; *p* = 0.04).

### 3.2. Changes in Salivary Alpha-Amylase Levels

**Figure 1:**
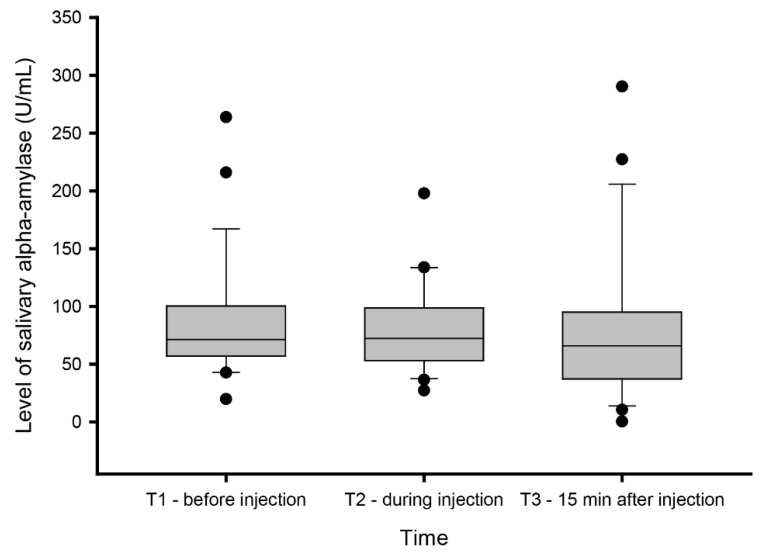
Salivary alpha-amylase levels. Levels of salivary alpha-amylase (sAA) measured before (T1), during (T2), and 15 min after (T3) experimentally induced muscle pain by injection of hypertonic saline 0.4 mL (56.5 mg/mL) into the masseter muscle.

Although the levels of sAA were higher before (87 ± 46.5 U/mL) than during (80.5 ± 37 U/mL) and after (81.7 ± 63.4 U/mL) experimentally-induced muscle pain, see Figure 1, the statistical analyses did not reveal any significant differences in sAA levels between samples collected before, during, or after experimentally-induced muscle pain (X_2_ = 1.68; *p* = 0.43).

### 3.3. Changes in Perceived Stress and Pain Level

There was a significant change in perceived stress (X_2_ = 29.7; *p* < 0.001) and level of pain (X_2_ = 49.0; *p* < 0.001) between the time points prior, directly after, and 15 min after experimentally-induced muscle pain. The post-hoc analysis with Bonferroni correction adjusted for multiple comparisons (*p* < 0.0017) showed a significant increase in perceived stress directly after pain was induced (Z = 3.57; *p* = 0.00035), which then significantly decreased 15 min after injection (Z = 4.01; *p* < 0.0001) and was at that time point (15 min after injection) normalized, showing no significant difference compared to baseline (Z = 2.20; *p* = 0.028).

**Figure 2:**
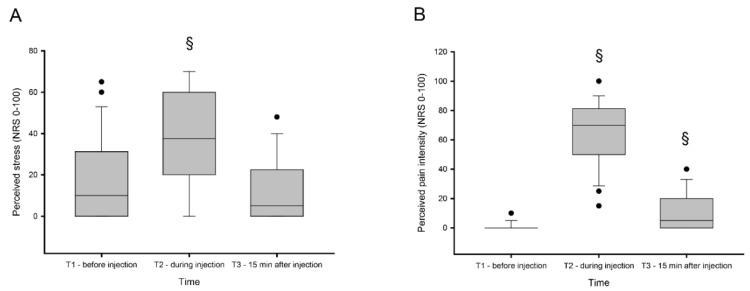
Perceived stress and pain. Perceived stress (**A**) and pain (**B**) levels measured on 0–100 numeric rating scale (NRS) before (T1), during (T2), and 15 min after (T3) experimentally-induced muscle pain by injection of hypertonic saline 0.4 mL (56.5 mg/mL) into the masseter muscle. § indicates significant difference when compared to baseline.

Perceived pain was similar to the perceived stress level, which also increased after experimentally-induced pain, and showed significant differences between all three collection points. Pain was most intense directly after injection (Z = 4.45; *p* = 0.0000) and showed a decreasing pattern, but did not return to baseline levels 15 min after injection (Z = 3.72; *p* = 0.0002) (Figure 2B).

### 3.4. Correlation Analysis

There were no significant correlations between sAA and pain and/or stress after adjustment for multiple comparisons. The sAA levels assessed prior to injection showed a moderately strong correlation to sAA levels directly after experimentally-induced muscle pain (r_s_ = 0.687; *p* < 0.0001). Furthermore, there were also a strong correlation between the perceived stress and level of pain during experimentally-induced muscle pain (r_s_ = 0.744; *p* < 0.0001)

## 4. Discussion

The main finding of this study was that experimentally-induced pain, i.e., acute pain, did not affect the levels of sAA, which seems to be in contrast to previous studies [20,21,22,23]. However, in contrast to the previous studies [5,8,20,21,22,23], this study used a short lasting, high intensity pain model [35,36,37,38]. This could have resulted in a pain state that was too short-lasting to activate the ANS, resulting in a secretion of sAA. On the other hand, the outcome of this study indicates that the used pain model seems to be an appropriate experimental model of stress [39].

sAA in the current study design, although non-significant, showed a tendency towards declining when acute pain was induced. The non-significant alternation in the sAA level during conditions of pain contrast previous studies investigating sAA in acute and chronic pan settings [7,20,21,22,23], but are in line with some other studies [5,8,18]. sAA has been suggested as an objective tool in pain perception, and thus far, only a few studies have examined the relationships between sAA and subjective pain perception [5,7,8,20,21,22,23,24]. Similar to this study design, two of the studies used experimentally controlled pain stimuli in healthy volunteers [7,24], while others observed the enzyme in patients with clinical pain states of an acute or chronic character [20,21,22,23]. Wittwer et al. studied the correlation between sAA, pain intensity rated on a visual analogue scale (VAS), and unpleasantness of experimental pain heat pain tolerance rated on a VAS. They found a significant correlation between VAS intensity and sAA levels as well as VAS reported level of unpleasantness. However, there was no significant correlation found between sAA activity and pain tolerance [7]. Another study by Van Stegeren et al. showed a significant increase of sAA concentrations in healthy subjects during a stressful task, but in contrast to our study, an additional painful task (cold pressor task) immediately increased the sAA, indicating that sAA has an immediate response to both stressful and painful stimuli [24]. Unlike our study, both these studies used temperature as stimuli, which might be a factor explaining why our study could not repeat the immediate response to the painful stimulus. Other studies have also revealed an association between sAA level and patients with chronic pain conditions [22,23]. One study, by Shirasaki et al., studied the correlation in patients with lower back and leg pain [23], while another study, by Arai et al., researched pain in cancer patients [22]. Shirasaki et al. showed a substantial correlation between sAA and pain intensity in patients suffering from chronic pain. The authors also concluded that the scale is sensitive enough to measure pain-induced psychological stress [23]. The study by Arai et al. showed a small but yet significant correlation between sAA activity and pain intensity in cancer patients [22]. However, there are numerous studies that have failed to show significant associations between sAA and pain intensity ratings [5,6,8,21]. Previous studies investigating sAA level in pain states have usually measured the amylase level in patients with pain lasting for several weeks, months, or even years. To our knowledge, this study is the first to observe changes in sAA during controlled, acute pain in an experimental setting. To compensate for the shorter pain duration compared to earlier studies, a higher dose of saline was used, compared to previous protocols [36,37,38], to induce a high intense but slightly prolonged pain sensation. Based on the study results, one may speculate that pain duration may have a stronger effect on the ANS and sAA secretion than we originally assumed, and that pain intensity has less significance. Pain duration may also influence the subjective perception of pain, and consequently, perceived stress.

Participants reported at baseline a low level of stress, but the levels increased significantly when pain was present and returned to similar levels as baseline when pain disappeared (Figure 2A.). Nociceptive stimuli are among the most powerful inducers of stress responses, and these results strongly indicate that injection of hypertonic saline increases psychological stress, and could thus be used as a suitable model of stress [39]. Animal studies in mammals have shown that a single injection of silane solution triggered a clear stress response on a systemic level, as well as in stress sensitive regions of the brain [40]. The subjective stress response reported by our participants were moderate and somehow weaker than reported by other stress models [39]. This may be due to the short duration of pain induced by the hypertonic saline injection or even by the study cohort that consisted of young university students that are used to be in stressful situations.

Hypertonic saline injections have been used extensively to induce muscle pain, since the quality of the pain mimics acute clinical muscle pain [36,37]. The participants in the study reported moderate pain intensity, with a peak after 30 s that spreads to adjacent regions and pain referral to other structures similar to several previous studies [36,37,38]. Hence, the quality of pain induced can be regarded as clinically relevant. The pain duration, however, was prolonged compared to previous findings with pain models using 0.2 mL of hypertonic saline [37,38]. Although pain intensity decreased significantly 15 min after injection, half of the participants still experienced a mild pain sensation, which was a result of the higher dose compared to previous studies [37,38]. Furthermore, based on previous studies indicating a rapid response to pain, within 3 min in the study from van Stegeren [24], one can discuss whether the lack of response is due to the stimulus type and not response time. Heat and cold activate thermal receptors on Aβ- or C-fibers, while algogenic substances (e.g., hypertonic saline) activate chemoreceptors on Aβ- or C-fibers [41].

The relationship between subjective pain and sAA are inconclusive. Some studies report a significant association, while others report no differences. Common in all studies is that they use perceived pain intensity as a tool to assess pain [7,8,20,22,23]. However, pain intensity itself may not be a reliable tool for assessing pain, especially for pain in the orofacial region [42,43], since other factors seems to play an important role in the individual’s perception of the pain experience [44]. Several studies have shown that subjective pain level poorly correlate to several biomarkers shown to be related to pain [26,45,46,47], but rather to different variables such as physical and psychological functioning [42,43,48,49,50]. Therefore, the multidimensional experience of pain [51], including not only intensity, but also the cognitive and affective component, should be taken into consideration in further studies [52,53,54,55,56]. The lack in most previous studies of evaluating physical and psychological parameters further strengthens the concept that the pain intensity itself might not affect sAA secretion. An additional problematic issue is the use of a single dimension scale when measuring pain intensity, such as the VAS. Although it has been the most used scale for pain measurement, it is not capable of identifying the multidimensional experience of pain, which is a limitation of most studies [42].

Several studies have reported higher pain intensity and a lower pain threshold and pain tolerance in women compared to men [57,58], which contradict the results in our study showing no significant differences between sexes. These results may be due to the rather young participants enrolled in the experiment without great experience of pain in earlier in life, and the large variation between participants in the relatively small study group erasing such differences.

A strength in our study design was the healthy homogeneous group and the controlled study environment. It was ensured via the anamnesis and the oral examination that the participants were healthy, and they were matched for age and gender to eliminate the influence of these factors on enzyme activity or reported stress levels. The pain model applied are well-known, and numerous studies have shown good reliability [36,37,38]. The experiment and sample collection followed the exact same protocol for all participates, and to avoid variation in sAA secretion, experiments were mainly conducted during the early afternoon.

Nevertheless, our findings should be interpreted within the context of certain limitations. For instance, the pain induced had a short duration, which does not accurately mirror pain under normal pathological circumstances, and the participants were aware of the experimental pain, which may have influenced the stress response. However, the experimental protocol was very rigorous, which should remove such a confounding factor. Furthermore, one factor that may have affected the sAA levels is the human salivary amylase gene, of which the expression is affected by the amount of starch in the diet [28]. When analyzing the results, it could also be observed that more than half of the participants still reported mild pain 15 min after the injection. This time interval was set based on earlier experiments showing that the pain experiences usually decreases 15 min after injection of the hypertonic saline into the masseter muscle [36,37]. It may be suggested to wait longer than 20–30 min for the pain to completely disappear until saliva is collected.

To conclude, this study could not show any association between the levels of sAA and perceived pain and or/stress, which agrees with the variety of different results regarding the levels of sAA. This indicates that it might not be pain itself that influences the levels of sAA, but instead the duration of the perceived discomfort/unpleasantness induced by pain. Furthermore, this study showed that even experimentally-induced pain induces stress, based on the significant strong correlation observed between perceived stress and pain intensity. Hence, this study indicates that experimentally-induced muscle pain could be used as a stress model. However, further studies need to confirm this outcome.

## Figures and Tables

**Table 1:** Descriptive data. Overview of the participants in the study. Questionnaire scores are presented as mean ± standard deviation or as median (interquartile range). There were no statistical differences between males and females in any of the variables, *p* > 0.05, Mann–Whitney *U*-test.

Variable	Males	Females	Statistics
Number of participants	13	13	
Age (years)	23.7 ± 2.7	23.8 ± 2.8	Z = 0.26; *p* = 0.80
Baseline pain (NRS)	0 (0)	0 (0)	Z = 0.31; *p* = 0.76
Baseline stress (NRS)	0 (20)	15 (38)	Z = 1.64; *p* = 0.09
PHQ-9 Score (0–27)	3 (3)	3 (6)	Z = −0.01; *p* = 0.94
PHQ-15 Score (0–30)	2 (3)	3 (3)	Z = 1.44; *p* = 0.15
STAI-STAIT	47 (7)	46 /7)	Z = −0.28; *p* = 0.78
STAI-TRAIT	46 (6)	46 (8)	Z = −0.36; *p* = 0.72
RPE (0–10)	2 (2)	2 (1)	Z = 1.38; *p* = 0.17

Abbreviation: NRS = Numeric Rating Scale; PHQ = The Patient Health Questionnaire; STAI = State and Trait Anxiety; STATE = current anxiety; TRAIT = anxiety in general; RPE = Rated Perceived Exertion.

## References

[1] Schumacher S., Kirschbaum C., Fydrich T., Ströhle A. (2013). Is salivary alpha-amylase an indicator of autonomic nervous system dysregulations in mental disorders?—A review of preliminary findings and the interactions with cortisol. Psychoneuroendocrinology.

[2] Fabian T.K., Hermann P., Beck A., Fejerdy P., Fabian G. (2012). Salivary defense proteins: Their network and role in innate and acquired oral immunity. Int. J. Mol. Sci..

[3] Nater U.M., Rohleder N., Gaab J., Berger S., Jud A., Kirschbaum C., Ehlert U. (2005). Human salivary alpha-amylase reactivity in a psychosocial stress paradigm. Int. J. Psychophys..

[4] Pedersen A.M., Bardow A., Jensen S.B., Nauntofte B. (2002). Saliva and gastrointestinal functions of taste, mastication, swallowing and digestion. Oral Dis..

[5] Silva Andrade A., Marcon Szymanski M., Hashizume L.N., Santos Mundstock K., Ferraz Goularte J., Hauber Gameiro G. (2018). Evaluation of stress biomarkers and electrolytes in saliva of patients undergoing fixed orthodontic treatment. Miner. Stomatol..

[6] Kobayashi F.Y., Gavião M.B.D., Marquezin M.C.S., Fonseca F.L.A., Montes A.B.M., Barbosa T.S., Castelo P.M. (2017). Salivary stress biomarkers and anxiety symptoms in children with and without temporomandibular disorders. Braz. Oral Res..

[7] Wittwer A., Krummenacher P., La Marca R., Ehlert U., Folkers G. (2016). Salivary Alpha-Amylase Correlates with Subjective Heat Pain Perception. Pain Med. (MaldenMass).

[8] Campos M.J., Raposo N.R., Ferreira A.P., Vitral R.W. (2011). Salivary alpha-amylase activity: A possible indicator of pain-induced stress in orthodontic patients. Pain Med. (MaldenMass).

[9] Nater U.M., Rohleder N. (2009). Salivary alpha-amylase as a non-invasive biomarker for the sympathetic nervous system: Current state of research. Psychoneuroendocrinology.

[10] Gilman S.C., Fischer G.J., Biersner R.J., Thornton R.D., Miller D.A. (1979). Human parotid alpha-amylase secretion as a function of chronic hyperbaric exposure. Undersea Biomed. Res..

[11] Wan C., Couture-Lalande M.E., Narain T.A., Lebel S., Bielajew C. (2016). Salivary Alpha-Amylase Reactivity in Breast Cancer Survivors. Int. J. Environ. Res. Public Health.

[12] Vineetha R., Pai K.M., Vengal M., Gopalakrishna K., Narayanakurup D. (2014). Usefulness of salivary alpha amylase as a biomarker of chronic stress and stress related oral mucosal changes—A pilot study. J. Clin. Exp. Dent..

[13] Granger D.A., Kivlighan K.T., el-Sheikh M., Gordis E.B., Stroud L.R. (2007). Salivary alpha-amylase in biobehavioral research: Recent developments and applications. Ann. N. Y. Acad. Sci..

[14] Van Stegeren A., Rohleder N., Everaerd W., Wolf O.T. (2006). Salivary alpha amylase as marker for adrenergic activity during stress: Effect of betablockade. Psychoneuroendocrinology.

[15] Noto Y., Sato T., Kudo M., Kurata K., Hirota K. (2005). The relationship between salivary biomarkers and state-trait anxiety inventory score under mental arithmetic stress: A pilot study. Anesth. Anal..

[16] Takai N., Yamaguchi M., Aragaki T., Eto K., Uchihashi K., Nishikawa Y. (2004). Effect of psychological stress on the salivary cortisol and amylase levels in healthy young adults. Arch. Oral Biol..

[17] Bosch J.A., de Geus E.J., Veerman E.C., Hoogstraten J., Nieuw Amerongen A.V. (2003). Innate secretory immunity in response to laboratory stressors that evoke distinct patterns of cardiac autonomic activity. Psycho. Med..

[18] Fischer S., Doerr J.M., Strahler J., Mewes R., Thieme K., Nater U.M. (2016). Stress exacerbates pain in the everyday lives of women with fibromyalgia syndrome—The role of cortisol and alpha-amylase. Psychoneuroendocrinology.

[19] Laurent H.K., Laurent S.M., Granger D.A. (2013). Salivary nerve growth factor reactivity to acute psychosocial stress: A new frontier for stress research. Psycho. Med..

[20] Ahmadi-Motamayel F., Shahriari S., Goodarzi M.T., Moghimbeigi A., Jazaeri M., Babaei P. (2013). The relationship between the level of salivary alpha amylase activity and pain severity in patients with symptomatic irreversible pulpitis. Restor. Dent. Endod..

[21] Bugdayci G., Yildiz S., Altunrende B., Yildiz N., Alkoy S. (2010). Salivary alpha amylase activity in migraine patients. Auto. Neurosci..

[22] Arai Y.C., Matsubara T., Shimo K., Osuga T., Ushida T., Suzuki C., Ohta A., Tohyama Y., Nishida K., Arakawa M. (2009). Small correlation between salivary alpha-amylase activity and pain intensity in patients with cancer pain. Acta Anaesthesiol. Scand..

[23] Shirasaki S., Fujii H., Takahashi M., Sato T., Ebina M., Noto Y., Hirota K. (2007). Correlation between salivary alpha-amylase activity and pain scale in patients with chronic pain. Reg. Anesth. Pain Med..

[24] Van Stegeren A.H., Wolf O.T., Kindt M. (2008). Salivary alpha amylase and cortisol responses to different stress tasks: Impact of sex. Int. J. Psychophysiol..

[25] Jasim H., Carlsson A., Hedenberg-Magnusson B., Ghafouri B., Ernberg M. (2018). Saliva as a medium to detect and measure biomarkers related to pain. Sci. Rep..

[26] Jasim H., Ghafouri B., Carlsson A., Hedenberg-Magnusson B., Ernberg M. (2020). Daytime changes of salivary biomarkers involved in pain. J. Oral Rehabil..

[27] Jasim H., Olausson P., Hedenberg-Magnusson B., Ernberg M., Ghafouri B. (2016). The proteomic profile of whole and glandular saliva in healthy pain-free subjects. Sci. Rep..

[28] Balodis I.M., Wynne-Edwards K.E., Olmstead M.C. (2010). The other side of the curve: Examining the relationship between pre-stressor physiological responses and stress reactivity. Psychoneuroendocrinology.

[29] Schiffman E., Ohrbach R., Truelove E., Look J., Anderson G., Goulet J.P., List T., Svensson P., Gonzalez Y., Lobbezoo F. (2014). Diagnostic Criteria for Temporomandibular Disorders (DC/TMD) for Clinical and Research Applications: Recommendations of the International RDC/TMD Consortium Network* and Orofacial Pain Special Interest Groupdagger. J. Oral Fac. Pain Headache.

[30] Kroenke K., Spitzer R.L., Williams J.B. (2001). The PHQ-9: Validity of a brief depression severity measure. J. Gen. Intern. Med..

[31] Maurer D.M. (2012). Screening for depression. Am. Fam. Phys..

[32] Kroenke K. (2006). Physical symptom disorder: A simpler diagnostic category for somatization-spectrum conditions. J. Psycho. Res..

[33] Spitzer R.L., Kroenke K., Williams J.B., Lowe B. (2006). A brief measure for assessing generalized anxiety disorder: The GAD-7. Arch. Intern. Med..

[34] Williams N. (2017). The Borg Rating of Perceived Exertion (RPE) scale. Occup. Med..

[35] Graven-Nielsen T., Arendt-Nielsen L. (2003). Induction and assessment of muscle pain, referred pain, and muscular hyperalgesia. Curr. Pain Headache Rep..

[36] Al Sayegh S., Borgwardt A., Svensson K.G., Kumar A., Grigoriadis A., Christidis N. (2019). Effects of Chronic and Experimental Acute Masseter Pain on Precision Biting Behavior in Humans. Front. Physiol..

[37] Louca S., Christidis N., Ghafouri B., Gerdle B., Svensson P., List T., Ernberg M. (2014). Serotonin, glutamate and glycerol are released after the injection of hypertonic saline into human masseter muscles—A microdialysis study. J. Headache Pain.

[38] Christidis N., Ioannidou K., Milosevic M., Segerdahl M., Ernberg M. (2008). Changes of hypertonic saline-induced masseter muscle pain characteristics, by an infusion of the serotonin receptor type 3 antagonist granisetron. J. Pain.

[39] Patchev V.K., Patchev A.V. (2006). Experimental models of stress. Dialogues Clin. Neurosci..

[40] Freiman S.V., Onufriev M.V., Stepanichev M.Y., Moiseeva Y.V., Lazareva N.A., Gulyaeva N.V. (2016). The stress effects of a single injection of isotonic saline solution: Systemic (blood) and central (frontal cortex and dorsal and ventral hippocampus). Neurochem. J..

[41] Graven-Nielsen T., Arendt-Nielsen L., Mense S. (2008). Fundamentals of musculoskeletal pain.

[42] Feine J.S. (2000). Treating chronic pain: How do we measure success?. New York State Dent. J..

[43] Ohrbach R., Dworkin S.F. (1998). Five-year outcomes in TMD: Relationship of changes in pain to changes in physical and psychological variables. Pain.

[44] Slade G.D., Ohrbach R., Greenspan J.D., Fillingim R.B., Bair E., Sanders A.E., Dubner R., Diatchenko L., Meloto C.B., Smith S. (2016). Painful Temporomandibular Disorder: Decade of Discovery from OPPERA Studies. J. Dent. Res..

[45] Schultz J., Uddin Z., Singh G., Howlader M.M.R. (2020). Glutamate sensing in biofluids: Recent advances and research challenges of electrochemical sensors. Analyst.

[46] Ernberg M., Goulet J., Velly A. (2017). Masticatory Muscle Pain Biomarkers. Orofacial Pain Biomarkers.

[47] Gerdle B., Ghafouri B., Ernberg M., Larsson B. (2014). Chronic musculoskeletal pain: Review of mechanisms and biochemical biomarkers as assessed by the microdialysis technique. J. Pain Res..

[48] Rudy T.E., Turk D.C., Kubinski J.A., Zaki H.S. (1995). Differential treatment responses of TMD patients as a function of psychological characteristics. Pain.

[49] Suvinen T.I., Reade P.C., Hanes K.R., Kononen M., Kemppainen P. (2005). Temporomandibular disorder subtypes according to self-reported physical and psychosocial variables in female patients: A re-evaluation. J. Oral Rehabil..

[50] Vlaeyen J.W., Morley S. (2005). Cognitive-behavioral treatments for chronic pain: What works for whom?. Clin. J. Pain.

[51] Villemure C., Bushnell C.M. (2002). Cognitive modulation of pain: How do attention and emotion influence pain processing?. Pain.

[52] Bushnell M.C., Duncan G.H., Hofbauer R.K., Ha B., Chen J.I., Carrier B. (1999). Pain perception: Is there a role for primary somatosensory cortex?. Proc. Natl. Acad. Sci. USA.

[53] Rode S., Salkovskis P.M., Jack T. (2001). An experimental study of attention, labelling and memory in people suffering from chronic pain. Pain.

[54] Haythornthwaite J.A., Benrud-Larson L.M. (2000). Psychological aspects of neuropathic pain. Clin. J. Pain.

[55] Meagher M.W., Arnau R.C., Rhudy J.L. (2001). Pain and emotion: Effects of affective picture modulation. Psychosom. Med..

[56] Keogh E., Ellery D., Hunt C., Hannent I. (2001). Selective attentional bias for pain-related stimuli amongst pain fearful individuals. Pain.

[57] Fillingim R.B. (2000). Sex, gender, and pain: Women and men really are different. Curr. Rev. Pain.

[58] Hashmi J.A., Davis K.D. (2014). Deconstructing sex differences in pain sensitivity. Pain.

